# Influence of Subsequently Applied Mechanical and Thermal Loads on Surfaces Ground with Mechanical Main Impact

**DOI:** 10.3390/ma14092386

**Published:** 2021-05-04

**Authors:** Rebecca Strunk, Florian Borchers, Brigitte Clausen, Carsten Heinzel

**Affiliations:** 1 Leibniz-Institute for Materials Engineering—IWT, Badgasteiner Str. 3, 28359 Bremen, Germany; borchers@iwt-bremen.de (F.B.); clausen@iwt-bremen.de (B.C.); heinzel@iwt.uni-bremen.de (C.H.); 2 MAPEX Center for Materials and Processes, Faculty of Production Engineering, University of Bremen, Badgasteiner Str. 1, 28359 Bremen, Germany

**Keywords:** surface integrity, process chain, internal material loads, grinding, mechanical impact, load case-based process adjustment, residual stresses

## Abstract

To generate advanced properties for the wear resistance and fatigue life of components and allow for an improved, application-oriented development of part specifications, a precisely tailored initial machining or manufacturing process is necessary. In addition, it is important to know how subsequent machining steps or operational loads affect the components’ condition. Residual stresses are a meaningful measurand for evaluating the modifications that a machining process induces into the material. The desired modifications should be specified regarding the final state for the required operational behavior. Thus, the stability of the modifications can be considered so that they can be beneficial in service. This investigation is part of fundamental research in the field of the Collaborative Research Center (CRC) “Process Signatures”. By applying defined selected loads, the effects on machined surface layers are investigated since machined components are exposed to further loads during use. For this reason, experimental process chains are applied in this work to grind-strengthened specimens as possible application cases and corresponding loads. These experimental process chains consist of defined mechanical and thermal loads, which are applied to the specimens using a thermal and mechanical testing system. Furthermore, it is investigated how these additional loads affect the modifications previously introduced by the grinding process. The influence of the additional loads is evaluated by using radiographic and electron microscopic examinations. It can be observed that the sequence, as well as the type of the applied loads, play a significant role in the development of the modifications.

## 1. Introduction

External loads caused by machining processes have had extensive consideration in scientific investigations since they have a significant impact on the properties of a final workpiece [1,2]. The workpiece surface is important since it defines many functional properties [3]. External loads may be process-specific, but they are the source of internal material loads, which are not process-specific. However, these loads are responsible for material modifications. The process impact by unified and coherent quantities, such as thermal and mechanical loads, can be described by correlating the process independent internal material loads with the material modifications [4,5]. This concept of a direct and process independent correlation of internal material loads and material modification is applied by the so-called process signatures [6,7]. The presented work investigates the changes that possible process chains might cause in the modifications evoked by previously applied machining processes. The aim of this fundamental research is to approach and understand the stability of modifications introduced by manufacturing processes and to support the evaluation of material simulations. Therefore the above-mentioned process chains consist of experimentally and simulative easy-to-apply thermal and mechanical loads. These thermal and mechanical loads, which are selected as subsequent loads after manufacturing, e.g., during operation, can be both applied separately and in combinations. In particular, the influence of the experimental process chains on the residual stress state is investigated. The investigated material is AISI4140 (42CrMo4) German-grade steel. The microstructural changes of the ferritic-perlitic annealed specimens of this investigation are discussed intensely by Ehle et al. [8].

Depending on material properties and physical effects, the mechanisms that lead to material modifications can vary, although resulting modifications such as residual stresses can be similar. The resulting material modification and its stability in further processing depends on the intensity and the relations of the internal material loads. Therefore, the mechanisms leading to material modifications should be considered in the interpretation of the results. For example, a change in surface hardness can be caused by thermal loads and phase transformation and mechanically induced strengthening effects. Changing the surface hardness in turn can either result in a positive or a negative effect on the workpiece properties, depending on the mechanism, the load quantity, and the desired properties of the finished component. High thermal loads induced by grinding processes can cause an increase in hardness [3,9]. If residual stress measurements on the surface show compressive residual stresses, it may be evaluated as a surface strengthening effect. If the measurements show tensile stresses, it may be assessed as surface damage [10,11]. 

The influence of residual stresses on the fatigue life of components has been the subject of many investigations, e.g., [12,13]. It is stated that residual stresses can be taken as additional mean stresses in fatigue prediction if they are mechanically stable [13,14,15]. Hirsch et al. [16] investigated the influence of tensile and compressive loads on a shot-peened aluminum-alloy and explained the results of his investigation using the surface-core model according to Vöhringer. Madariaga et al. [17] investigated the evolution and the stability of residual stresses in Inconel 718 under quasi-static loading. In this study, the surface-core model of Vöhringer was used to explain the relaxation behavior of the residual stresses. By investigating the effect of mechanical and thermal loads on residual stresses in shot-peened AISI4140, Holzapfel et al. [14] concluded that the mechanisms of residual stress relaxation due to mechanical and thermal loading can be considered as separate processes. Furthermore, the effect of bending on shot-peened surfaces is discussed, stating that the effects depend on the initial state of the material [14,18]. 

The mechanism behind the mechanical relaxation of residual stresses is the transformation of elastic deformations into microplastic deformations. This can be explained due to dislocation movements that cause the dislocations to rearrange or annihilate each other [15]. According to the literature on thermal residual stress relaxation, the speed-determining process for the reduction of residual stresses is the diffusion-controlled climbing of edge dislocations [19]. Macro-residual stresses are reduced faster than microresidual stresses [15,19,20,21]. A mechanism change occurs at a temperature of 300 °C. While the dominant mechanism for T < 300 °C is creep, it climbs the edge dislocations at T > 300 °C [22].

To gain a comprehensive understanding of processes, the specific implied material loads and their impacts must be taken into account. However, the measurement and validation of internal material loads within a machining process are challenging. Hence, applying defined material loads, correlated by measured corresponding modifications, can be used to gain information about the mechanisms as well as interdependencies of previous modifications and support the simulation of additional processing steps. Therefore, the research approach of this work is to apply simple but defined experimental process chains to manufactured specimens. The process chains consist of defined mechanical and thermal loads that are applied to the specimens using a thermal and mechanical testing system. The influence of the additional loads is evaluated by using radiographic and scanning electron microscopy. 

## 2. Materials and Methods 

### 2.1. Specimens

**Figure 1:**
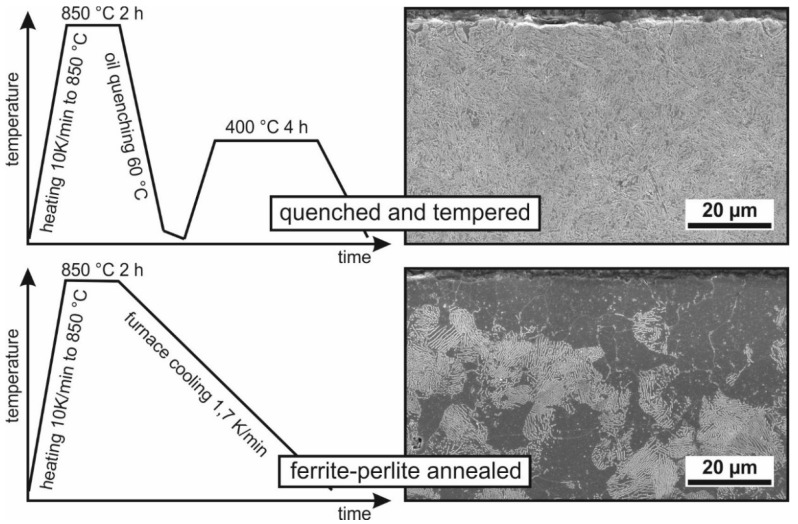
Heat treatment parameters and SEM-micrographs of the microstructure.

**Figure 2:**
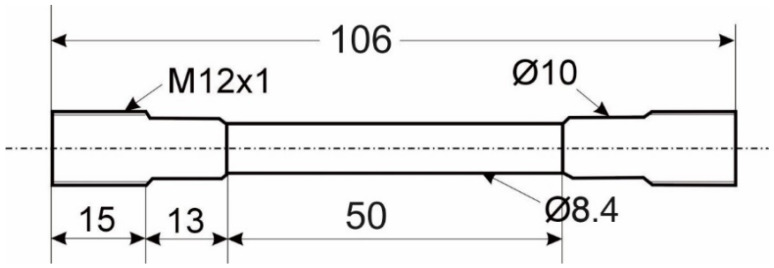
Design of the applied specimens.

The specimens are made from an AISI4140 (42CrMo4) German-grade steel. All specimens were manufactured from the same batch of material. The initial state was set by means of soft machining (turning) and two different heat treatments, resulting in two microstructural states. The quenched and tempered (QT) state shows a very fine-grained, needle-like tempered martensite. The ferritic-perlitic annealed (FP) specimens have a fine grain structure consisting of ferrite and perlite grains. The heat treatment parameters and scanning electron microscope (SEM)-micrographs in secondary electron contrast of the initial microstructures are shown in Figure 1. The geometry of the specimens is shown in Figure 2. Threads are needed for the specimen grips and prevent the specimen from slipping when clamped in the thermal and mechanical testing system. The geometry of the specimen was determined by the dimensions of the specimen grips of the thermal and mechanical testing system, Gleeble 3500 (Dynamic Systems, New York, NY, USA). Therefore, no standard tensile specimens were used. The specimens were manufactured with an allowance of 0.4 mm in diameter for the grinding process. The final diameter of the tensile specimens was 8.0 mm. 

### 2.2. Grinding Process

**Figure 3:**
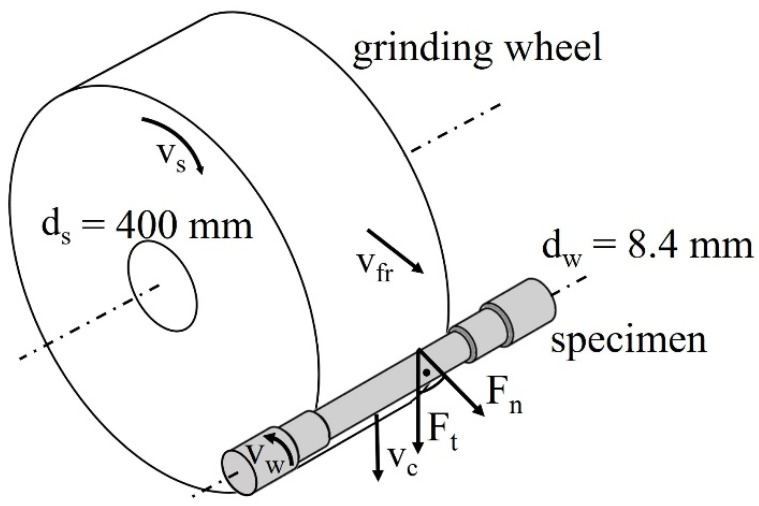
Sketch of grinding process with the grinding wheel and specimen.

Grinding was selected as a process with a high impact on the functionality of the workpieces due to its characteristics as a finishing process. It is utilized as a representative manufacturing process. The used machine tool was a Studer S41 (Steffisburg, Switzerland) high-power grinding machine with integrated piezoelectric force measurement. To gain good signal accuracy, eight three-axis sensors were integrated below the workpiece spindle and tailstock, so that the measurement was implemented directly into the force flow on the workpiece. The grinding process kinematics with the corresponding process parameters, and the forces in the normal direction (F_n_) and in tangential direction (F_t_), are illustrated in Figure 3. 

For the investigations, a grinding process with a pronounced mechanical impact was applied. Due to the mechanical effect on the surface integrity, it is referred to as “grind-strengthening”. The process was conducted as a cylindrical plunge grinding with a rotating tool and linear radial feed speed v_fr_. Due to symmetric material removal along the grinding width, the axial forces were negligible. The mechanical impact of the grinding process is based on the shift of grinding energy from material removal to a relatively large amount of plastic material deformation due to the process adjustment. A relatively low chip thickness and rather low cutting speeds are prerequisites for that effect [23]. To implement the plunge grinding over the whole surface length, a grinding wheel width of b_w_ = 50 mm was used to cover the length with the diameter of d_w_ = 8.4 mm, as shown in Figure 2 and Figure 3. The final diameter of that section of the workpiece after grinding was d_w_ = 8.0 mm. Despite the workpiece diameter and restrictions of the rotational speed of the spindle, the grinding velocity was selected relatively low (v_c_ = 12 m/s). A vitrified bond corundum grinding wheel was utilized with a medium grain size to reduce thermal and promote mechanical load impacts. For the experiments, a grinding oil was applied as a metalworking fluid with a defined flow rate. Fluid supply conditions were also adapted to the process demands and kept constant for every experiment. The experimental parameters are shown in Table 1. 

Due to the width of the grinding wheel (b_w_ = 50 mm), which was also the intended contact width, no axial movement was applied. The edges of the grinding wheel had a process-related radius on the outsides with contact to the workpiece surface. Hence there were no process-related sharp edges on the investigated area of the specimens. However, the mechanical impact for the selected parameters could be determined by the measurements and were sufficient for the investigations. After finishing of each single specimen, a dressing procedure was conducted to reduce influences of wear and alteration of the grinding wheel surface between the processes. It was conducted with a form roll and was performed twice after each specimen. The dressing parameters are shown in Table 1. 

**Figure 4:**
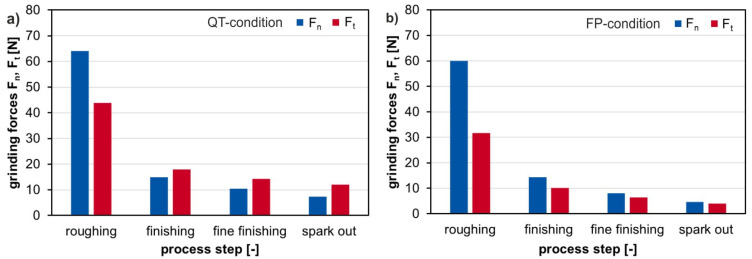
Force measurement results for (**a**) a specimen in QT-condition (**b**) a specimen in FP-condition.

The grinding process was separated into three process stages, rough grinding, finishing and fine finishing. A sparkout time of t_s_ = 4 s was applied with a radial feed speed of v_fr_ = 0 mm/min to generate an even and finished surface result. As expected, due to the selected process parameters, the first stage has the largest external material load (see grinding forces) with a descending tendency during the rough grinding stage. The rather soft FP material has smaller maximum resulting grinding force values than the harder surface of the QT-condition. Figure 4 shows the measured maximum force values, F_n_ and F_t_, for the mentioned process stages for one workpiece and each workpiece material state.

### 2.3. Application of Defined Loads after Grinding

The surface layers produced by grind-strengthening were subjected to defined mechanical and thermal loads to experimentally reproduce simple process chains or application cases with specific loads. A thermal and mechanical testing system (type Gleeble 3500, Gleeble, New York, NY, USA) was utilized to apply the selected loads to the specimens. The testing system allows an exact adjustment and measurement of the applied loads. The tensile tests were performed at a strain rate of ε; = 0.001 1/s and were strain controlled. The elongation is controlled by a laser extensometer. 

**Figure 5:**
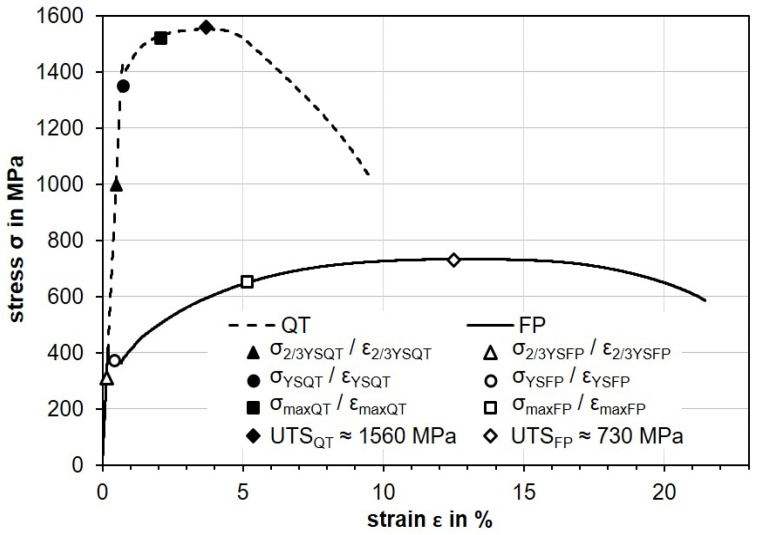
Results from tensile tests conducted on AISI 4140 in the two heat treatment conditions, FP and QT, with indicated chosen mechanical loads and the ultimate tensile strength (UTS_FP_ and UTS_QT_).

The selection of the mechanical loads was based on the stress–strain curves determined for the two initial material conditions FP and QT of the AISI4140 (42CrMo4); see Figure 5. The material properties of the initial conditions and the three selected mechanical loads are shown in Table 2. The lowest applied stress was in the range of about two-thirds of the respective yield point (defined as σ_2/3YSFP_ or σ_2/3YSQT_) as an example for nominal elastic deformation. The second mechanical load was chosen at the respective yield point (defined as σ_YSFP_ or σ_YSQT_) to allow the first plastic deformation. The third load was selected so that a maximum load (defined as σ_maxFP_ and σ_maxQT_) could be applied without exceeding the ultimate tensile strength (respectively UTS_FP_ or UTS_QT_). These values were selected in order to ensure uniform plastic deformation in both heat treatment conditions. In order to apply the selected loads, the tensile test was stopped when the corresponding stresses were reached and the specimens were relieved subsequently. 

An annealing treatment of one hour at 700 °C was selected exemplarily as the thermal load for both material conditions. It was chosen assuming that existing residual stresses will be relieved by mechanisms such as recrystallization or recovery at this temperature [24]. The thermal load is applied by conductive heating with a heating rate of 5 K/s. An overview of the selected loads is shown in Table 2. 

For the investigation of the influence of simple defined mechanical or thermal loads on the generated surface layer, a test program consisting of different combinations of the selected loads was defined. The test program consisted of five different loading concepts. The specimens were loaded with:

1. A mechanical load (σ_maxFP_ or σ_maxQT_).
2. A sequence of an initial mechanical load (σ_maxFP_ or σ_maxQT_) followed by a thermal load (1 h at 700 °C).
3. A thermal load (1 h at 700 °C).
4. A sequence of an initial thermal load (1 h at 700 °C) followed by a mechanical load (σ_maxFP_ or σ_maxQT_).
5. A simultaneous combination of the mechanical (σ_maxFP_ or σ_maxQT_) and the thermal load (1 h at 700 °C).

Two specimens were tested for each selected load or load sequence.

### 2.4. Measurement of Surface Modifications

The material modifications were investigated in detail by X-ray diffraction (XRD) measurements by means of residual stresses and full width at a half-maximum of the measured intensity peaks (FWHM). To evaluate the influence of the mechanical and thermal impact on the material modification, the specimens were examined in the conditions provided after grinding as well as after the application of additional mechanical and thermal loads. The measurement of the residual stresses and FWHM at the surface as well as for the depth profiles was carried out in the middle of each specimen. The change in the residual stress on the surface and residual stress depth profiles, can be understood as an indicator for the stability of the surface modification. 

**Figure 6:**
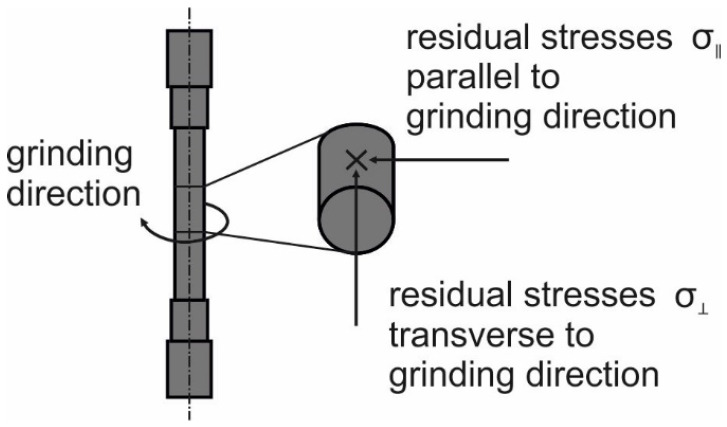
Grinding direction and directions of the measured residual stresses on the specimens.

The residual stress measurements were conducted by XRD stress analysis, which analyzed a single measurement position with θ-2θ diffractometer (GE Inspection Technology, Seifert XRD MZ VI E, XRD Eigenmann GmbH, Schnaittach-Hormersdorf, Germany) with vanadium-filtered Cr-Kα radiation. For the measurements, a primary beam diaphragm of 1 mm diameter and a gas-filled, linear position-sensitive detector of the Photron-X Miostar I type was used. The stress calculation was performed using specific material parameters based on the examined steel alloy with sin^2^ψ-method [25]. The measurements were made in two directions with respect to the grinding direction. A schematic depiction of the directions of the grinding process and the residual stresses are shown in Figure 6. As shown in Figure 2, the grinding direction was transverse to the longitudinal direction of the specimen. The choice of the reference system parallel to grinding direction leads to the fact that the residual stresses transverse to the grinding direction (σ_⊥_) are in tensile direction. To illustrate the respective directions of the residual stresses, a sketch was attached to the diagrams.

For the measurements in depth direction, material was removed by electrolytic polishing accordingly. Since all residual stress measurements were conducted with the previously mentioned setup and application, the measurement errors and the specific influence of the measurement system can be assumed as nearly constant. In addition to the investigation of the residual stresses, the FWHM is also considered in this work. 

The FWHM indicates the appearance of local changes in the lattice plane distances. There are many causes for local changes in the lattice plane distances, such as alloying elements, precipitations or microstresses. Microstrains can emerge from either thermal or plastic deformation [25]. Generally, an increase in the amount of grain boundaries and dislocations cause a broadening of the interference peak [26,27]. Therefore, the FWHM can also be used to evaluate strain hardening effects [28,29]. The work hardening caused by grind-strengthening appears relatively close to the surface (approximately 100 µm or less), so that hardness measurements are useful to a limited extent. In this case, the FWHM can be used to assess the microstructural changes.

The scanning electron microscopic investigations were carried out at a Vega II XLH Tescan. For an overview of the microstructural changes, deep-etched micrographs from cross-sections of the grind-strengthened specimens were viewed with the use of secondary electron contrast. The cross-sections from the specimens were taken from the middle of the specimens. This approach allowed a higher magnified view on the microstructure. More detailed investigations of the specimens in FP-condition can be found in Ehle et al. [8].

## 3. Results

### 3.1. Influence of Different Mechanical Loads on Grind-Strengthened Surface Layer Modifications

**Figure 7:**
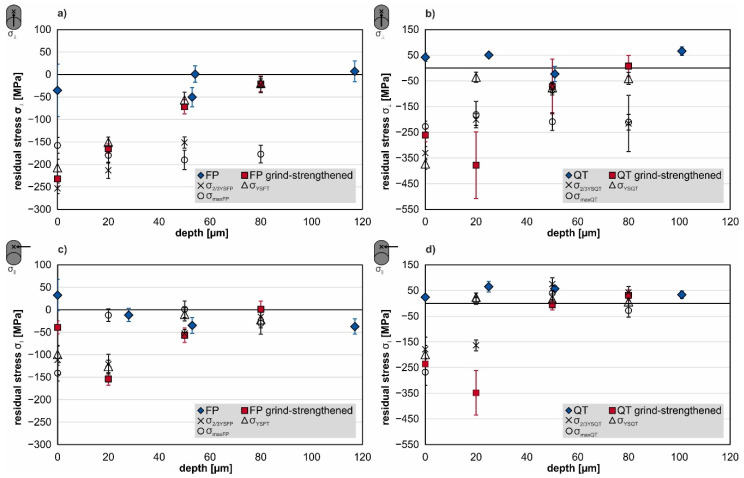
Residual stress depth profiles transverse (upper images) and parallel (lower images) to grinding direction of grind-strengthened specimens in FP-condition (**a**,**c**) and QT-condition (**b**,**d**).

The measured residual stress depth profiles after grind-strengthening and subsequent loading with different mechanical loads are shown in Figure 7. The error bars represent the standard deviation of two measured specimens. All specimens show very low residual stresses over the entire depth in the initial heat-treated state. The values fluctuate with ±50 MPa around 0 MPa. 

The FP-annealed grind-strengthened specimens show the highest residual compressive stresses transverse to the grinding direction on the surface; see Figure 7a. The other variants show maximum residual stresses beneath the surface. The values at 20 µm beneath the surface are similar for different measuring directions in the FP-condition and respectively in the QT-condition. The absolute values of the residual stresses decrease until the level of the residual stresses of the initial material is reached at a depth of about z = 80 µm.

An additional load of σ_2/3YSFP_ shows an increase in the value of residual compressive stresses transverse to the grinding direction up to a depth of z = 50 µm in the FP-condition. At a depth of z = 80 µm, the value of the residual stresses lies within the range of the initial FP material. The other variants show a slight increase of the compressive stresses at the surface. The residual stresses parallel to the grinding direction in the FP material show only slightly lower values in the further depth; see Figure 7c. The QT-condition shows a significant reduction at 20 µm depth; see Figure 7b,d. Applying σ_YSFP_ to the FP-condition results in measured values (marked with open triangles) similar to that of the grind-strengthened specimen (red squares). In the QT-condition, transverse to grinding direction the surface value increases; see Figure 7b. At 20 µm depth and to further depths the residual stresses are almost extinguished. With the application of the respective σ_max_ (marked with circles), a change in depth profile appears. The compressive residual stresses transverse to the grinding direction on the surface are lower than after grind-strengthening; see Figure 7a,b. The other measuring points remain at a constant level (σ_⊥FP_ = −175 MPa, σ_⊥QT_ = −200 MPa. The measurement ends at a depth of z = 80 µm. However, the affected depth is higher than for the other treatments. The compressive residual stresses parallel to the grinding direction (σ_‖_) show the maximum value at the surface and are already on the level of the initial material (either FP- or QT-condition) at 20 µm depth after application of the respective σ_max_. 

### 3.2. Full Width at Half Maximum Depth Profiles

**Figure 8:**
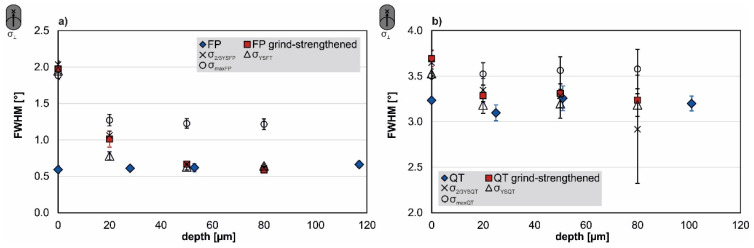
Full width at half maximum transverse to grinding direction in (**a**) FP- and (**b**) QT-condition.

The following section describes the full width at half maximum of the intensity peaks at the residual stress measurement determined after grind-strengthening and the subsequently applied mechanical loads. For comparison, the FWHM of the initial FP- or QT-conditions is displayed in Figure 8. The depths profiles of the FWHM in both measurement directions do not differ significantly. Therefore only the depth profiles transverse to grinding direction are described here. The blue rhombuses indicate the profile for the initial material. The FWHM of the AISI 4140 in FP-condition is about 0.6°; see Figure 8a. The FWHM in the QT-condition is approximately 3.2 to 3.3°; see Figure 8b. The red squares symbolize the FWHM after grind-strengthening. In both heat treatment conditions the FWHM broadens at the surface. The FWHM increases to about 2° at the surface in the FP-condition. At a depth of 20 µm, the FWHM is still about 1°. The surface value of the FWHM increases to about 3.7° in the QT-condition. Already at a depth of 20 µm, the values reach the level of the initial conditions. A load of the respective σ_2/3YS_ (cross markers) or a load of the respective σ_YS_ (open triangles) did not significantly influence the FWHM. A plastic deformation due to the respective σ_max_ implies no measurable change of the surface value but increases the FWHM in further depth. The value of the FWHM of the FP-condition is increased to 1.2°. The FWHM of the QT-condition is increased up to 3.5°. 

### 3.3. Surface Residual Stresses after Different Loads and Loading Sequences

**Figure 9:**
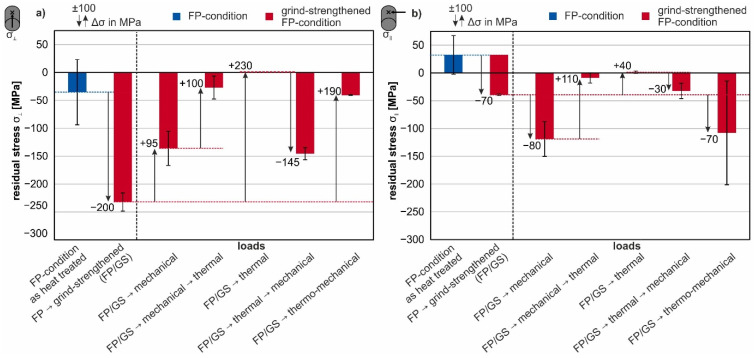
Surface residual stresses after grind-strengthening and various loads for the material in FP-condition (**a**) transverse to grinding direction (**b**) parallel to grinding direction.

**Figure 10:**
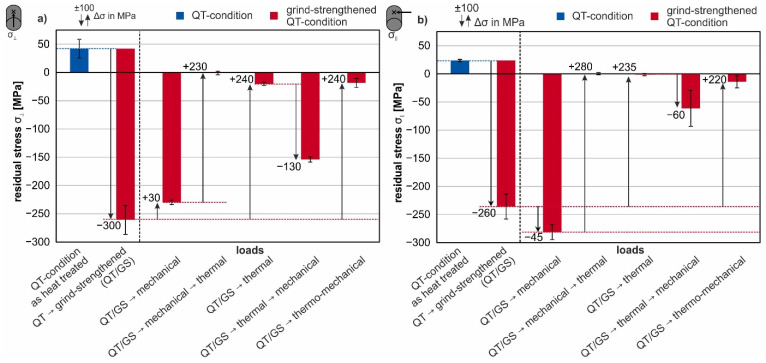
Surface residual stresses after grind-strengthening and various loads for the material in QT-condition (**a**) transverse to grinding direction (**b**) parallel to grinding direction.

The following Figure 9 and Figure 10 show the surface residual stresses of the grind-strengthened specimens in the FP- and QT-condition. The blue columns show the initial surface residual stresses of either the FP- or QT-condition before grinding or application of additional defined material loads. The first red column shows the surface residual stresses after grinding in direct relation to the initial residual stresses. The following red columns in the diagrams show the results after applied loads and therefore, the effects of the different loads and load sequences on the surface residual stresses of the grind-strengthened condition. The changes in the residual stresses compared to the grind-strengthened or otherwise influenced conditions are illustrated by arrows pointing in the direction of the change. The amount of the change of the residual stresses is written at the tip of the arrows. 

The error bars indicate the standard deviation of the measurements. Each specimen was measured once, and the deviation was derived from the measurement of two specimens of the same condition. The standard deviation of the specimens in the initial heat treatment condition was calculated from the measurement of three nominal equal specimens for each steel condition of different batches. The occurring standard deviation is composed of measurement system errors, deviations due to the curved surface, roughness of the surface, and material inhomogeneities. The influence of surface effects on the standard deviation can be estimated by the comparison of surface values and values measured in depth (e.g., Figure 7).

The influence of the different loads or load sequences on grind-strengthened specimens in FP-condition is shown in Figure 9. The surface residual stresses transverse to grinding direction are depicted in Figure 9a. As expected, grind-strengthening causes compressive residual stresses at the surface due to the mechanical impact of the grinding process [30,31].

A mechanical load of σ_maxFP_ reduces the compressive residual stresses in comparison to the initial grind-strengthened condition. The thermal load leads to a complete elimination of the residual stresses on the surface (column 5 in Figure 9a). The residual stress resulting from a sequence of an initial mechanical load followed by a thermal load (column 4 in Figure 9a) is σ_⊥_ = −27 MPa. This is a first indication that the type and order of the subsequent loads can have an influence on the final state and the material or workpiece behavior. 

However, the total amount of resulting residual stress after the loading sequence of mechanical and thermal loads is low and within a typical error range of ±30 MPa in the measured surface stresses. Still, a similar trend is observed for the application of thermal and mechanical loads in the opposite order—the application results in compressive residual stresses of σ_⊥_ = −145 MPa. The combined thermo-mechanical load and its influence on the residual stresses is represented in the last column of Figure 9a. The combination of both loads causes a reduction of the residual stresses to approx. σ_⊥_ = −40 MPa.

The results of the measurement parallel to the grinding direction (σ_‖_) are illustrated in Figure 9b. In the initial condition, low tensile stresses are measured on the surface. The compressive residual stresses in the grinding direction after grind-strengthening are less pronounced than transverse to the grinding direction.

The influence of the mechanical load σ_maxFP_ on the residual stresses parallel to the grinding direction is illustrated by the third column of Figure 9b. The measured values for the mechanical, the thermal and the combined thermo-mechanical treatment are similar to the values in transverse direction, which implies that the initial residual stresses in the surface are compensated by these loads. In contrast, the sequence of an initial thermal load followed by a mechanical load (column 6 in Figure 9b) leads to smaller compressive residual stresses as in transverse direction (column 6 in Figure 9b). The stress in the grinding direction is in good accordance with Poisson’s ratio, about three times smaller than in transverse direction. 

The results for the grind-strengthened 42CrMo4 steel in the QT-condition are depicted in Figure 10. The blue column illustrates the surface residual stress state of the initial QT-condition. A slight tensile residual stress state is present on the surface. The second column of Figure 10a illustrates the residual stresses on the surface after grind-strengthening transverse to grinding direction of the QT-condition. A pronounced compressive residual stress state can be found. After applying the mechanical load σ_maxQT_ the compressive residual stresses are slightly reduced. The reduction of compressive residual stress is less significant than for the material in FP-condition.

The thermal load (column 5 Figure 10a) triggers an almost complete relief from residual stresses on the surface. A sequence of an initial mechanical load followed by a thermal load lead to a full relaxation of the residual stresses in both measuring directions. After the reversed sequence of loads (initial thermal load followed by a mechanical load), residual compressive stresses of about σ_⊥_ = −153 MPa occur; see column 6 in Figure 10a. The combined loads lead to a massive relaxation of the compressive residual stresses; see column 7 in Figure 10a.

The residual compressive stress state in grinding direction is more pronounced for the QT-condition than in the FP-condition; see Figure 10b. The third column shows the resulting residual stresses after the application of the mechanical load of σ_maxQT_. The plastic deformation transverse to the measurement direction increases the amount of compressive residual stresses compared to the grind-strengthened state. However, it is known from the depth profiles (Figure 7) that this is only a surface effect. The thermal load almost completely relieves the residual stresses; see column 5 of Figure 10b. If the loads are applied in reverse, the resulting residual stresses are compressive with the amount of σ_‖_ = −61 MPa. The thermomechanical load results in a very small amount of compressive residual stresses, as can be seen in the last column of Figure 10b. These results are comparable to the results parallel to the grinding direction (Figure 10a). 

### 3.4. Scanning Electron Microscope Micrographs of the Ground Specimens in FP- and QT-Condition

**Figure 11:**
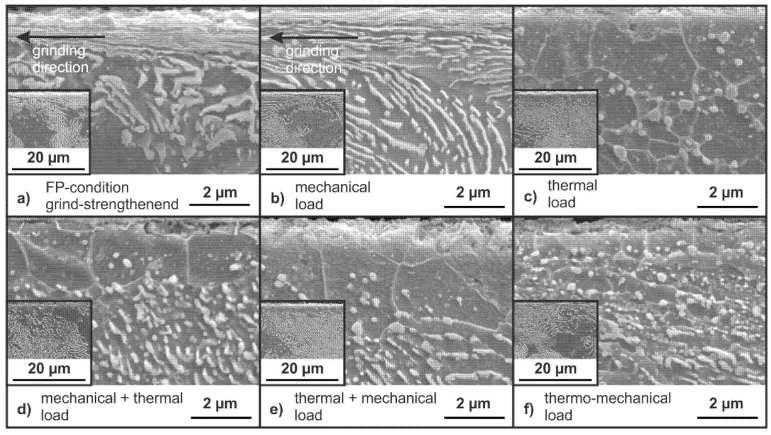
SEM-micrographs of the FP-condition with small cutouts in lower magnification for an impression of the microstructure (**a**) as ground and after the application of (**b**) a mechanical load (σ_maxFP_); (**c**) a sequence of an initial mechanical load (σ_maxFP_) followed by a thermal load (1 h at 700 °C); (**d**) a thermal load (1 h at 700 °C); (**e**) a sequence of an initial thermal load (1 h at 700 °C) followed by a mechanical load (σ_maxFP_); (**f**) a simultaneous combination of the mechanical (σ_maxFP_) and the thermal load (1 h at 700 °C).

**Figure 12:**
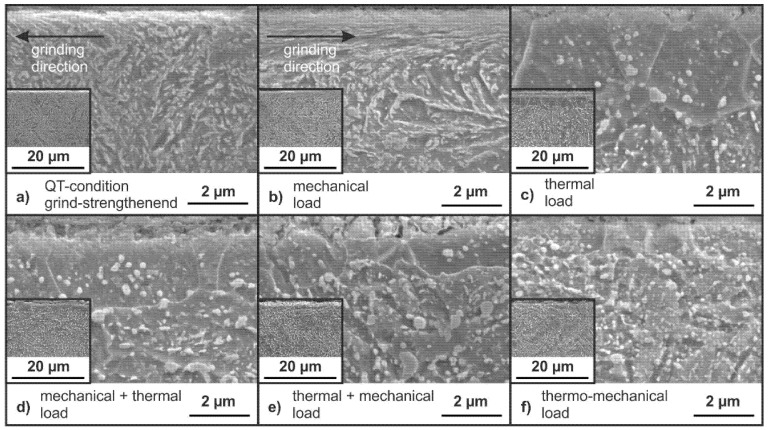
SEM-micrographs of the QT-condition with small cutouts in lower magnification for an impression of the microstructure (**a**) as ground and after the application of (**b**) a mechanical load (σ_maxQT_) (**c)** a sequence of an initial mechanical load (σ_maxQT_) followed by a thermal load (1 h at 700 °C) (**d**) a thermal load (1 h at 700 °C) (**e**) a sequence of an initial thermal load (1 h at 700 °C) followed by a mechanical load (σ_maxQT_) (**f**) a simultaneous combination of the mechanical (σ_maxQT_) and the thermal load (1 h at 700 °C).

The following SEM-micrographs display the changes in the microstructure after grinding with mechanical main impact (see Figure 11a and Figure 12a) as well as the microstructure after the application of the loading concepts (see Figure 11b–f and Figure 12b–f). The surface of the specimens is on the upper side of the micrographs. The microstructure near the surface clearly shows deformed grains as a visible modification of the grind-strengthening process, as shown in Figure 11a and Figure 12a. These deformations are oriented in grinding direction as the microstructure is deformed in grinding direction due to the process and material removal. This orientation is far more pronounced in the FP-condition (Figure 11a) than in the QT-condition (Figure 12a). The FP microstructure is deformed up to a depth of about z = 10 µm; see cutout in Figure 11a. 

Near the surface, the perlite lamellae are compressed, which can be seen distinctly in Figure 11a. Underneath the deformed area, the undeformed FP microstructure is visible; see Figure 11a’s small cutout. The microstructure is not visibly influenced by the application of a mechanical load (σ_maxFP_); see Figure 11b. The selected thermal load results in a visible change in the near-surface microstructure, as depicted in Figure 11c and Figure 12c. For both material conditions, FP- and QT-condition, a seam of regular grains is visible on the surface. In both heat treatment conditions, the thickness of this seam is about 4 to 5 µm. For the FP-condition, the cementite lamellae formed into spherical cementite particles. These are visible underneath the presumably recrystallized grains, as can be seen in Figure 11c. In both conditions, spherical cementite particles are embedded in the regular ferrite grains. Underneath the seam of regular grains, the initial microstructures of the FP-condition on the one hand and on the other hand, the initial QT-condition remain; see small cutouts Figure 11d and Figure 12d.

The microstructure after a sequence of an initial mechanical load (σ_maxFP_) followed by a thermal load (1 h at 700 °C) is depicted in Figure 11d. A seam of regular grains is visible at the surface. Compared to the microstructure of a specimen loaded with a sequence of an initial thermal load (1 h at 700 °C) followed by a mechanical load (σ_maxFP_) shown in Figure 11e the grains in Figure 11d seem to be smaller. The effect of a thermo-mechanical load is shown in Figure 11f resulting in a different microstructure in the surface area, where spherical cementite particles are visible in a ferritic matrix. The cutout (Figure 11f) shows that the microstructure of the initial FP material is visible underneath.

In the QT-condition the grain deformation is not as pronounced as in the FP-condition; see Figure 12a. The microstructure is only slightly deformed, and the deformed zone can be assumed to about 1 µm. The deformed microstructure from the grinding process is oriented in grinding direction, which in this case is to the left side in the micrographs. Below the small deformed area near the surface the initial QT microstructure is visible; see the small cutout in Figure 12a. 

The microstructure after application of a mechanical load (σ_maxQT_) does not differ much from the as ground condition, as compared in Figure 12a,b. The deformed area at the surface looks a little more pronounced. The effect of a sequence of an initial mechanical load (σ_maxQT_) followed by a thermal load (1 h at 700 °C) is shown in Figure 12d and results in a seam of regular grains slightly smaller in comparison to the seam of grains in Figure 12c. The microstructure in Figure 12e is comparable to Figure 12d with a visible seam of regular grains near the surface; but in this case, with cracks starting from the surface. The application of a thermo-mechanical load results in a microstructure with visible spherical cementite particles in a tempered matrix near the surface. The cutout shows the initial QT material underneath the influenced area.

## 4. Discussion

To discuss the first three columns in Figure 9 and Figure 10, the depth profiles shown in Figure 7 should be taken into account. The residual stress state in the heat-treated condition fluctuate with ±50 MPa around 0 MPa. Since the average grain size is about 20 µm in the FP microstructure (see SEM-micrographs of the cross-sections in Figure 10), the residual stress can be assumed to change from grain to grain. Due to the very large plastic deformation of the outer surface layer, the compressive stresses after grind-strengthening transverse to the grinding direction reach their maximum value at the surface. In this direction, an elongation of the outer surface due to grinding must have occurred. 

The QT-condition and the measurement direction parallel to grinding direction in the FP-condition show the highest compressive stresses directly underneath the surface at 20 µm. Shear stresses due to the acting forces are assumed to elongate the material locally to generate these residual stresses. The measurable influence on the residual stresses only extends to a depth of 40 µm, corresponding to the enhanced FWHM. The strengthening effect of the grinding process may have been even more pronounced with different setup conditions, such as a larger diameter of the specimens or a lower cutting speed v_c_, respectively. The deformation of the whole specimen in the tensile test leads to an additional enhanced deformation in the surface region due to the easier deformability of the free surface. 

**Figure 13:**
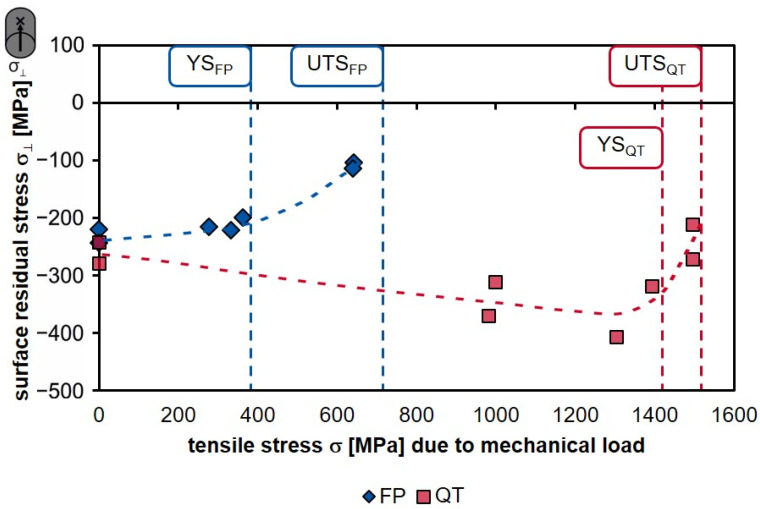
Surface residual stress transverse to grinding direction of grind-strengthened specimens as a function of the load stress in the tensile test.

In Figure 13, a plot of the residual stresses σ_⊥_ on the surface transverse to grinding direction is shown as a function of the applied tensile stress. The compressive residual stresses decrease in value when the mechanical load (tensile stress) reaches the yield strength. The decrease is even more pronounced when the tensile load exceeds the yield point. Together with the residual stress depth profiles (σ_⊥_), it becomes clear that the reason for the relaxation of the surface residual stresses is the total deformation of the specimens, and therefore the rearrangement of the residual stresses in the radial direction (see Figure 7). 

When the load exceeds the yield point in both material conditions, a plateau of compressive residual stress in the measured depth is formed, whereas the residual stresses on the surface are lowered in value; see Figure 7. The extent of rearrangements is a result of the alignment of the grinding process, different directions of the residual stresses and the applied mechanical load. Therefore, the intended utilization of defined manufactured surfaces with special modifications requires the knowledge of the direction of operational loads in the application. Hence, the material’s characteristics can be improved by considering the load amount and direction as well as the material modification due to that load. 

On the other hand, the rearrangement of the residual stresses is a result of the differences in the local strengths of the steel. Due to the grind-strengthened surface, the finished surface layer has a higher strength than the core material. To underline this, the FWHM are also considered. For both material conditions, a broadening of the peak occurs at the surface and the near-surface region, which can be interpreted as an indicator for the strengthening of the steel [28], as can be seen in Figure 8. Due to these different local strengths, the surface-core model according to Vöhringer is useful to explain the reduction of the residual stresses [15,17]. On the one hand, the core yields when strained with a force above the yield point. On the other hand, yielding begins later in the strengthened surface layer and the surface layer yields less than the core. After relief, the plastically deformed (“longer”) core elongates elastically the surface layer and therefore relaxes the compressive residual stresses. In the FP-condition, according to Ehle et al. [8], the dislocation density within the subgrains is reduced which indicates the occurrence of dynamic recovery as an additional mechanism behind the reduction of the residual stresses. The QT-condition has a higher dislocation density due to the heat treatment, which is further increased by grind-hardening. Again the FWHM depth profiles underline this interpretation. Additionally, after grind-strengthening the FWHM is increased compared to the initial QT-condition; see Figure 8.

The changes of the residual stresses parallel to grinding direction (σ_‖_) are in good accordance with those transverse to grinding direction (σ_⊥_). The effects are less pronounced since the stress perpendicular to the loading direction is reduced according to Poisson’s ratio.

The selected thermal load leads to a complete elimination of the residual stresses resulting from the grinding process in the surface. This is caused by recrystallization of the surface layer, as shown in the micrographs in Figure 11d and Figure 12d. New regular grains are visible in the surface regions. There is a significant difference between the degree of deformation visible in the grind-strengthened microstructure in the QT- and FP-conditions (see the small cutouts in Figure 11a and Figure 12a) because the matrix in the FP-condition is softer and more easily deformable than the matrix in the QT-condition. After annealing, the effect of recrystallization is similar in both microstructures (see Figure 11d and Figure 12d). The resistance to plastic deformation of the QT material is higher than that of the material in the FP-condition, but obviously the same amount of energy has been stored in the deformed surface.

The effect of different load sequences emphasizes that the order in which the loads are applied to the material is significant. The application of the load sequence of an initial mechanical load followed by a thermal load results in a small proportion of residual compressive stresses remaining in both measurement directions. In the QT-condition, the proportion of remaining residual compressive stresses is lower than in the FP-condition. This might be due to the bigger value of dislocation in the QT-condition from the beginning. The residual stress state is erased due to the application of the thermal load, and corresponding microstructural changes appear.

As expected, if the load sequence is reversed to a sequence of an initial thermal load followed by a mechanical load (σ_maxFP_ or σ_maxQT_), a new residual compressive stress state is obtained. The residual compressive stresses generated in this way differ only slightly in value for both material states and are lower than after grind-strengthening. The almost identical residual stress state at the surface after the application of that load sequence is due to the fact that the specimens in the two microstructural states should now have a similar surface condition due to the preceding thermal load. The occurrence of compressive residual stresses in the former “residual stress free” surface due to tensile strain indicates that the recrystallized surface has a lower yield strength than the core. Additionally, the free surface can be deformed easier. Both assumptions lead to a better deformability of the surface, leading to compressive stresses in the surface after relief.

A simultaneous application of a mechanical and a thermal load results in completely different modifications. In case of the thermo-mechanical load, different material modification mechanisms superpose each other. The thermo-mechanical load causes only a small amount of compressive residual stresses. The almost complete relaxation of the residual stresses is the result of the additional tempering process. The remaining stresses cannot exceed the warm yield point at 700 °C due to the slow cooling after thermo-mechanical treatment, working like a stress-relieving treatment. The high temperature facilitates the movement of dislocations and thus the healing of lattice defects caused by deformation or stress. Figure 9 and Figure 10 show that the remaining residual stresses are lower in the QT-condition than in the FP-condition. The differences in the residual stress states of the two microstructures are not very pronounced. Dynamic recrystallization is the predominant mechanism, as shown in the investigations of Ehle et al. [8]. 

The results of the application of different mechanical loads underlines that a relaxation of residual stresses through mechanical loads only occurs when the mechanical load reaches at least the local yield strength of the material. The depth profiles of the residual stresses and the FWHM are in good accordance. A comparison clearly shows that the residual stresses at the surface are reduced or rearranged, but the FWHM at the surface are not affected. This indicates that reduction of the residual stresses might be caused by a rearrangement of dislocations as the sub-grain boundaries are sharper in case of the application of σ_maxFP_ (see Ehle et al. [8]). On the other hand, the FWHM seems less affected because the microstructural differences at the surface are not very pronounced [8] although the residual stresses are reduced. The broadening of the FWHM near the surface on the one hand and the broader FWHM in greater depth, on the other hand, prove the deformation of the material. The broadening at the surface is caused by the grinding process. It indicates a change in the microstructure. In this case, it is a strengthening of the material caused by work hardening because of the formation of dislocations and the implicated deformation of the material. The formation of a plateau in the depth profile (see Figure 8) with the values of the FWHM higher than the initial material proves the entire deformation of the specimens. 

## 5. Conclusions

In our investigation, we demonstrated how subsequent material loads in the form of a defined mechanical and thermal load, respectively, as well as combinations and sequences thereof, can take effect on the surface layer modified from preceding grinding with a main mechanical impact. The new aspect of this research is the analysis of the effects on surface and subsurface caused by different external loads and the comparison of the occurring modifications to understand the mechanisms and interactions. 

The presented results have shown that even discrete single loads can cause changes in the initially desired modifications:

- Subjecting the ground specimens to different mechanical loads in the order of two-thirds of the respective yield strength, close to the respective yield strength, and close to the respective ultimate tensile strength confirmed that the yield strength is a critical parameter for applied mechanical loads since plastic deformation occurs when this parameter is exceeded. The results show that the residual stresses were lowered the closer the selected load was to the local yield strength.
- Subjecting the selected thermal results in a complete relaxation of the residual stresses caused by the grinding process due to recrystallization and resulting in the same microstructure at the surface.

In good agreement with the state of the art, it was found that the different specific loads caused different modifications and thus different changes in the surface and subsurface state. By applying the selected loads in different sequences on the ground surfaces of the specimens and by the evaluation of the resulting residual stresses as well as the FWHM, it was shown that the order in which the loads are applied is of significant importance. As:

- Subjecting a sequence of an initial mechanical load (σ_maxFP_ or σ_maxQT_) followed by a thermal load (1 h at 700 °C) to the ground specimens results in decreased residual stress at the surface.
- Subjecting a sequence of an initial thermal load (1 h at 700 °C) followed by a mechanical load (σ_maxFP_ or σ_maxQT_) to the ground specimens results in a similar residual stress state for both investigated material conditions and similar microstructures near the surface.
- Subjecting a simultaneous combination of the mechanical (σ_maxFP_ or σ_maxQT_) and the thermal load (1 h at 700 °C) to the ground specimens result in only a small amount of residual stress due to the tempering process. The dominant mechanism is dynamic recrystallization according to Ehle et al. [8].

The investigation revealed that the direction of the application or impact with regard to residual stresses induced by grinding is of importance either. Since the external mechanical load was applied to the specimens through tensile stress and the grinding direction was transverse to it, it was possible to compare the resulting surface stresses and acting forces according to their directions. The results show the stability of the modifications introduced by grinding with a mainly mechanical impact regarding the amount and direction of the subsequently applied loads. For machined components, it is particularly important to know the stability of residual stresses induced by specific manufacturing processes, since these contribute to the functionality.

The results of this study are particularly appropriate to evaluate the simulation of the application of mechanical, thermal and thermo-mechanical loads, since the simplicity and definition of application avoids the overlook of boundary conditions. The evaluation is suitable for checking which microstructural effects have to be taken into account or can be neglected also for the simulation of more complex conditions.

Future work should take lower temperatures and cyclic loads into consideration. Thus, the stability of the material (surface) modifications and the mechanisms of dynamic loading will be investigated more detailed. The intensity and the build-up and reaction of the modifications can help to extend the understanding of the mechanisms between internal material loads and the effect on material modifications such as residual stresses. In addition, comparisons to other mechanisms and the effects of the modifications e.g., generated by other manufacturing processes with varying load intensities, should be considered.

## Figures and Tables

**Table 1:** Process parameters for the applied grinding process with mechanical main impact.

Workpiece		Grinding Wheel	
material	AISI4140 (42CrMo4)	specification	9A60H16V
diameter	d_w_ = 8.4 mm	diameter	d_s_ = 400 mm
tangential feed speed	v_w_ = 1.8 m/s (down-hill grinding)	width	b_w_ = 50 mm
		wheel speed	v_s_ = 13.8 m/s
**dressing parameters**		**process parameters**	
depth of cut in dressing	a_e,d_ = 20 µm	depth of cut	a_e_ = 150 µm/40 µm/10 µm
overlap ratio	U_d_ = 3	radial feed speed	v_fr_ = 2.0 mm/min/0.4 mm/min/0.2 mm/min
speed ratio	q_d_ = 0.6	cutting speed	v_c_ = 12 m/s
		spark out time	t_s_ = 4 s

**Table 2:** Mechanical properties of the material conditions and applied loads on the ground specimens in the thermal and mechanical testing system.

	Material Condition	
	FP	QT
yield strength (YS)	380 MPa (YS_FP_)	1420 MPa (YS_QT_)
ultimate tensile strength (UTS)	730 MPa (UTS_FP_)	1560 MPa (UTS_QT_)
mechanical loads	tensile test up to	
	σ_2/3YSFP_ ≈ 300 MPa (ε_2/3YSFP_ ≈ 2/3 YS_FP_)	σ_2/3YSQT_ ≈ 990 MPa (ε_2/3YSQT_ ≈ 2/3 YS_QT_)
	σ_YSFP_ ≈ 370 MPa (ε_YSFP_ ≈ YS_FP_)	σ_YSQT_ ≈ 1350 MPa (ε_YSQT_ ≈ YS_QT_)
	σ_maxFP_ ≈ 650 MPa (ε_maxFP_ = 5%)	σ_maxQT_ ≈ 1520 MPa (ε_maxQT_ = 2%)
thermal load	annealing for 1 h at 700 °C	
thermo-mechanical load	tensile test at 700 °C up to	
	σ_maxFP_ ≈ 650 MPa (ε_maxFP_ = 5%)	σ_maxQT_ ≈ 1520 MPa (ε_maxQT_ = 2%)

## Data Availability

Data sharing is not applicable to this article.
